# MIGRATION, STRESS AND SUCCESSFUL AGING AMONG GRANDPARENTS: A MIXED METHODS STUDY

**DOI:** 10.1093/geroni/igad104.3469

**Published:** 2023-12-21

**Authors:** Yue Hu, Helene Fung

**Affiliations:** The Chinese University of Hong Kong, Hong Kong, Hong Kong; The Chinese University of Hong Kong, Hong Kong, Hong Kong

## Abstract

**Objectives:**

the present work aims to explore the stress and coping related to successful aging among migrant and non-migrant grandparents.

**Methods:**

both qualitative and quantitative methodologies were employed. Twenty-one grandparents (12 migrants, median age = 59 years old, 14 females) in mainland China were interviewed about the definition of successful aging, as well as stress and coping related to successful aging based on ecological systems theory. Another 303 grandparents (92 migrants, mean age 61.2 ± 4.8, 158 females) were recruited to complete some questionnaires about the intensity of grandparenting, proactive coping, successful aging, as well as some demographic information (e.g., migration, socioeconomic status).

**Results:**

the interview data revealed that all grandparents valued harmonious family relationships most. Migrant grandparents uniquely faced stressors in different ecological systems, i.e., hardships in their close relationships, inadequate social welfare, and uncertainty in aging preparation. Regarding coping strategies, migrant grandparents uniquely employed avoidance coping strategies when facing intergenerational conflicts and preparing for their later life, as compared with non-migrant grandparents, in the process of perceiving successful aging. The quantitative study found that intensive grandparenting activated more proactive coping thus establishing a successful late life, which only existed among non-migrant grandparents.

**Discussion:**

Migration has a detrimental effect on older adults’ successful aging via imposing extra pressure on grandparents’ life, and/or damaging their ability to proactively cope with potential challenges.

